# Recurrent Hematochezia Due to a Rectal Fistula Caused by a Left Internal Iliac Artery Aneurysm

**DOI:** 10.7759/cureus.79483

**Published:** 2025-02-22

**Authors:** Hiroya Masuda, Yu Yamamoto, Ryusuke Ae

**Affiliations:** 1 Department of General Medicine, Sunagawa City Medical Center, Sunagawa, JPN; 2 Division of Public Health, Center for Community Medicine, Jichi Medical University, Shimotsuke, JPN; 3 Division of General Medicine, Jichi Medical University, Shimotsuke, JPN

**Keywords:** aneurysm, arterio-enteric fistula, contrast-enhanced computed tomography, gastrointestinal bleeding, hematochezia

## Abstract

Arterio-enteric fistulas (AEFs) are a rare cause of gastrointestinal bleeding involving direct communication between an artery and the intestinal tract. The duodenum is the most common fistula site for AEFs, but none of the rectal cases are known. We present the case of an elderly man with hematochezia due to a rectal fistula caused by a left internal iliac artery aneurysm, which was detected on contrast-enhanced computed tomography (CT), showing typical fistula formation and ectopic gas images. This case highlights the importance of considering the occurrence of rare but potentially fatal AEFs in elderly patients with hematochezia and that the timely performance of contrast-enhanced CT is critical for an accurate diagnosis.

## Introduction

Arterio-enteric fistulas (AEFs) are a rare cause of gastrointestinal bleeding, characterized by a direct connection between an artery and the intestinal tract [1]. They are an uncommon but critical cause of gastrointestinal bleeding that requires prompt recognition and management. Fistulas commonly form in the esophagus, duodenum, and small intestine, with rectal fistulas being rare [2]. AEFs are classified as primary arterio-enteric fistulas (PAEFs), which develop spontaneously due to aneurysm erosion, infection, or inflammation, and secondary arterio-enteric fistulas, which occur as a complication of prior aortic surgical intervention, such as graft placement. PAEFs are often associated with aneurysms, particularly in elderly patients with comorbid atherosclerosis [3,4]. Diagnosis is usually challenging due to nonspecific symptoms, and the classical triad of gastrointestinal bleeding, abdominal pain, and a palpable mass is often unreliable [3]. Early diagnosis and timely treatment are critical because untreated AEFs have an approximately 100% mortality rate [2]. Given their high fatality rate, AEFs should always be considered in patients with unexplained gastrointestinal bleeding, particularly those with a history of abdominal aortic aneurysm or prior aortic surgery. A definitive diagnosis is confirmed by contrast-enhanced computed tomography (CT), revealing a fistula between the artery and the adjacent gastrointestinal tract or ectopic gas in the aneurysm; however, these findings are rarely observed [5]. In this report, we present the case of an elderly man with persistent hematochezia due to a rectal fistula caused by a left internal iliac artery aneurysm, which showed typical fistula formation and ectopic gas images on contrast-enhanced CT.

## Case presentation

An 88-year-old man presented with recurring hematochezia over the past five months. He had no abdominal pain, nausea, vomiting, anal pain, stomatitis, fever, weight loss, or symptoms other than the chief complaint. Although he had a stroke 30 years ago, he did not have a regular hospital visit. He had no history or symptoms indicative of chronic inflammation, infection, or underlying collagen vascular disease, including vasculitis. He did not regularly take any prescribed oral medication. He had no history of alcohol consumption, smoking, or allergy and had never undergone colorectal cancer screening. His family history was unremarkable. He lived alone and was independent in his daily activities. However, he experienced severe hearing loss, relied primarily on writing, and had poor comprehension due to dementia. His body temperature was 36.4°C, blood pressure was 125/97 mmHg, pulse rate was 128 beats per minute, and respiratory rate was 16 breaths per minute. On physical examination, the eyelid conjunctiva showed no pallor. Heart sounds were clear, with no murmurs, and normal alveolar breath sounds were heard. The abdomen was flat, soft, and non-tender, with no detectable abdominal masses. Rectal examination identified an obstructive, non-pulsating mass at 1-3 o’clock, resulting in brick-like bloody stools. Laboratory tests revealed macrocytic anemia, mildly elevated CRP level, and elevated erythrocyte sedimentation rate (Table 1).

**Table 1:** Laboratory test results on the initial hospital visit

Test	Results	Reference Values
White Blood Cell Count	7,300/µL	4,000–9,000/µL
Neutrophils	67%	45–70%
Lymphocytes	21%	30–45%
Monocytes	7%	3–10%
Eosinophils	4%	2–10%
Basophils	1%	0–2%
Hemoglobin	10.9 g/dL	13.5–18 g/dL
Mean Corpuscular Volume	105 fL	85–100 fL
Platelet Count	165,000/µL	150,000–350,000/µL
Total Protein	7.5 g/dL	6.7–8.3 g/dL
Albumin	3.2 g/dL	4.0–5.0 g/dL
Aspartate Aminotransferase	34 U/L	13–33 U/L
Alanine Aminotransferase	17 U/L	8–42 U/L
Lactate Dehydrogenase	276 U/L	119–229 U/L
Creatinine	0.72 mg/dL	0.6–1.1 mg/dL
Blood Urea Nitrogen	17.5 mg/dL	8.0–22 mg/dL
Sodium	140 mEq/L	138–146 mEq/L
Potassium	4.5 mEq/L	3.6–4.9 mEq/L
Chloride	102 mEq/L	99–109 mEq/L
C-reactive Protein	0.67 mg/dL	<0.3 mg/dL
Erythrocyte Sedimentation Rate	81 mm/hour	1–7 mm/hour

**Figure 1:**
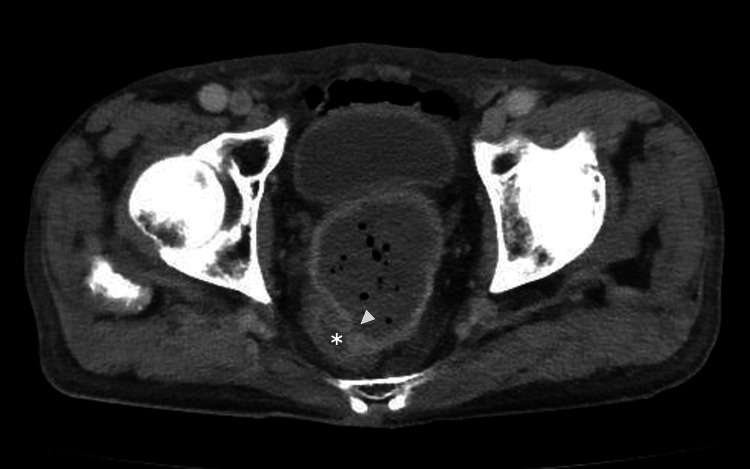
Contrast-enhanced CT indicates a rectal fistula caused by a left internal iliac artery aneurysm, characterized by ectopic gas within a giant aneurysmal sac and fistula formation (arrowhead) in the rectum (asterisk)

The differential diagnosis for this patient's recurrent bloody stools included diverticulosis, angiodysplasia, ischemic colitis, rectal ulcer, and colorectal cancer. However, the presence of a palpable rectal mass on digital examination, despite the absence of other symptoms, made evaluating neoplastic lesions the primary clinical priority. Given the patient's elevated risk of bowel obstruction and the need for urgent assessment, as well as the need to evaluate local tumor extension and possible metastasis to lymph nodes or distant organs, contrast-enhanced CT was chosen over endoscopy due to its superior capability to provide comprehensive staging while minimizing procedural invasiveness. The CT revealed a 60-mm left internal iliac artery aneurysm with ectopic gas, forming a fistula with the rectum (Figure 1).

Based on the CT results, the final diagnosis was a rectal fistula caused by a left internal iliac artery aneurysm. Because the risk of aneurysm rupture, fistula enlargement, or bacteremia due to the endoscopic procedure was deemed too high, we decided against performing a colonoscopy. 

Although surgical treatment was initially considered, the patient’s advanced age and cognitive decline led to the choice of conservative treatment with antibiotics in accordance with an infectious aortic aneurysm. The patient received outpatient follow-up care but ultimately died approximately three months after the diagnosis.

## Discussion

AEFs represent a medical condition in which an abnormal connection forms directly between an artery and the intestinal tract [1]. The common sites of fistula formation are the esophagus, duodenum, and small intestine [2]. In the literature, there have been few cases of fistula formation with the rectum among those with AEF. This report presents a case of a left internal iliac artery aneurysm that developed a fistula with the rectum, exhibiting characteristic AEF features on contrast-enhanced CT. Furthermore, this case emphasizes the significance of including AEFs in the differential diagnosis when evaluating elderly patients presenting with hematochezia.

Lower gastrointestinal bleeding is frequently attributed to various etiologies, with diverticulosis (15%-55%) being the predominant cause, followed by ischemic colitis (6%-18%), anorectal disorders (hemorrhoids, anal fissures, rectal ulcers 6%-16%), neoplasia (3%-11%), angiodysplasia (0%-3%), post-polypectomy bleeding (0%-13%), inflammatory bowel disease (2%-4%), and radiation colitis (1%-3%) [6]. In contrast, AEFs are considered a relatively rare etiology, accounting for only 0.18% of gastrointestinal bleeding cases [1]. The mean age of presentation for AEF patients is 64 years, with clinical manifestations typically including gastrointestinal bleeding that may persist from three days to five months or beyond [3,4]. The classic triad of AEFs includes gastrointestinal bleeding, abdominal pain, and a pulsatile mass [4]. The detection of a palpable pulsatile mass on physical examination strongly suggests the presence of a large aneurysm, making a distinctive feature rarely observed in other causes of gastrointestinal bleeding. While abdominal pain alone does little to narrow the differential diagnosis, the detection of a pulsatile mass should significantly heighten suspicion for AEFs in patients presenting with hematochezia. The mortality rate for untreated AEFs is approximately 100%, underscoring the critical importance of its prompt and accurate diagnosis followed by timely intervention [2].

Clinicians must first exclude common causes of gastrointestinal bleeding such as diverticulosis, angiodysplasia, ischemic colitis, rectal ulcer, and colorectal cancer. A definitive diagnosis of AEFs is based on the identification of a fistula connecting an artery to the adjacent gastrointestinal tract or the presence of ectopic gas within the aneurysm on contrast-enhanced CT; however, these findings are rarely observed [5]. The extravasation of the contrast material from the aorta into the intestinal lumen is also an infrequent occurrence [7]. Exploratory laparotomy is highly effective at identifying a fistula (sensitivity 91%-100%) [8], although this procedure is highly invasive. Endoscopy, widely regarded as the gold standard for diagnosing gastrointestinal bleeding, has certain limitations when used for AEF detection. Specifically, its diagnostic accuracy for AEFs ranges from 25% to 40% [3]. Therefore, patients with persistent abdominal pain and gastrointestinal bleeding should be suspected of having AEFs and may be required to undergo contrast-enhanced CT, even if the endoscopic findings appear unremarkable. In addition, in frail elderly patients who cannot tolerate procedures or in cases of suspected intestinal obstruction, contrast-enhanced CT is a useful initial examination as it is less invasive, quicker, and capable of evaluating vascular lesions, including AEFs.

In this case, the patient exhibited hematochezia persisting over five months, representing a notably protracted diagnostic timeline when compared with previously documented cases [9]. The anatomical positioning of the rectum within the confined pelvic space, coupled with the presence of a giant aneurysm, created conditions conducive to prolonged friction between these structures. Given these unique anatomical and temporal factors, it is plausible that the fistula formation in this case might have been more readily observable than in previous cases.

Conditions associated with ectopic gas or abnormal arterial communication on CT, such as infective aortic aneurysm and arteriovenous malformation, can mimic the imaging findings of AEFs and should be carefully distinguished. Infective aortic aneurysms typically present with air or fluid collections around the vessel [10]. Arteriovenous malformation appears as homogeneously enhancing dilated vascular structures with attenuation similar to the feeding and draining vessels [11]. Conversely, key features that differentiate AEFs include an adherent bowel loop to the aneurysm sac, ectopic gas located between the bowel and aneurysm, or extravasation of aortic contrast material into the enteric lumen, indicating a direct arterial connection [7]. These findings are critical for the definitive diagnosis of AEFs. 

The primary cause of AEFs is predominantly an aneurysm, and an AEF typically arises from friction between the outermost layer of an artery and the adjacent bowel wall. Although these aneurysms can occur in isolation, they frequently coexist with atherosclerosis, accounting for 60%-85% of cases, especially in the elderly [3]. When an AEF originates from an abdominal aortic aneurysm, the mass is palpable in 25%-70% of cases [12-14]. In contrast, aneurysms in aortic branches, such as the internal iliac artery, are not externally palpable and often go unnoticed by patients. Consequently, the initial manifestation of an aneurysm might be its rupture or symptoms related to fistula formation, such as gastrointestinal bleeding. Therefore, it is crucial to consider an AEF as a potential diagnosis for hematochezia in the elderly, despite its uncommon occurrence.

The optimal management of AEFs focuses on controlling hemorrhage, repairing bowel damage, and eradicating infection. However, the literature lacks direct comparisons between conservative treatment and surgical or endovascular interventions. In frail elderly patients, conservative treatment is often preferred due to the high risks associated with surgery. Nevertheless, without surgical intervention, mortality from hemorrhage and sepsis remains exceedingly high. Even with surgery, mortality rates approach 50% [15]. Endovascular treatment has demonstrated lower in-hospital mortality than open surgery, though the two-year survival rates are comparable for both approaches [16]. Therefore, early diagnosis and timely treatment are essential before the disease’s progression.

## Conclusions

We experienced a case of an elderly man with an AEF involving the rectum, diagnosed by the typical findings of contrast-enhanced CT. Clinicians are encouraged to consider fatal AEFs as a differential diagnosis for hematochezia, especially in the elderly. The timely performance of contrast-enhanced CT, an essential diagnostic tool, is critical for an accurate diagnosis of AEFs.
